# ‘Unburden us and them’: encountering ‘the other’ in meetings between Bosnian genocide survivors and Dutch UN veterans

**DOI:** 10.3389/fsoc.2025.1543549

**Published:** 2025-03-28

**Authors:** Siri Driessen, Jeannette van Brenk, Nicole L. Immler, Eric Vermetten

**Affiliations:** ^1^Department of Citizenship and Humanisation of the Public Sector, University of Humanistic Studies, Utrecht, Netherlands; ^2^Department of Psychiatry, Leiden University Medical Center (LUMC), Leiden, Netherlands

**Keywords:** return trip, Srebrenica genocide, Bosnia and Herzegovina, veterans, moral imagination, narratives, Dutchbat, encounter

## Abstract

Recently, the Dutch government granted ‘Dutchbat 3’ veterans and their partners the opportunity to return to Srebrenica and its surroundings, where they had been located up until the genocide of 1995. An important part of these return trips is dedicated to on-site meetings with women survivors of war and genocide. These encounters are thought to encourage more dialogue, mutual understanding, and an engagement with ‘the other’s’ points of view, with the aim of transforming the relationship between the participants. However, the conditions needed to make these encounters equal and meaningful are not yet fully understood. Levelling the playing field in encounters implies an ‘unlearning’ of earlier acquired perspectives, narratives, and worldviews, and involves mutual openness and respect. The success of an encounter is dependent on the willingness of visitors and hosts to think and do differently. This might be challenging in a context in which the memory of past conflict is highly gendered, polarized and politicized. By better grasping whether and in what ways encounters with ‘the other’ might become meaningful, it could be possible to design and implement these encounters accordingly. In this article, we aim to identify the conditions needed to enable or disable such encounters. Based on ethnographic research of survivors and veterans, we ask: which conditions need to be met to make the encounters meaningful for the participants? We argue that their current form has potential, but that, to be successful, more attention is needed to better understand what engaging with ‘the other’ really requires: it means being ready to ‘restory’ the past and be open to different perspectives. Our research shows that this is not easily done: dominant narratives feed into dichotomous memory cultures, causing people to fall back into old patterns, despite the fact that both groups had suffered from very similar forms of institutional neglect. To redress this the conceptualization of the encounters and return trips would need to be carefully considered.

## Introduction

*“Alright. Now, the question here for everyone is, are we ready to sit down all together, that there be veterans… those soldiers… and that they speak about their lives and that we speak about our lives (…). And you know why that’s important: to relax this entire situation. That you get an answer to the [questions] that you have. And that they hear the real truth.”*

It may seem a real challenge to ask women survivors of the Srebrenica genocide to meet Dutch UN (Dutchbat 3) veterans—those who were deployed to the enclave and its surroundings right up until the genocide of more than 8,000 Bosniaks committed by Bosnian-Serb (para)military troops in July 1995. Encounters between survivors and veterans have been rare: despite a few attempts, the contact between these groups has remained minimal. This situation changed recently when the Dutch government announced that they would facilitate return trips to Srebrenica for these veterans, in which they visit the sites that were meaningful to them during the deployment. An important part of these trips is dedicated to encounters between veterans and women survivors, that are hosted by the Potočari Memorial Centre and the Bosnian Women’s Association Snaga Žene [women’s power] in Tuzla. In these meetings, veterans, their partners, and survivors are encouraged to listen to each other, talk about how they continued their lives after the war, and ease the often tense relationship that exists between them.^1^ But achieving this has notable challenges. This is understandable given the sensitive context of the Srebrenica genocide and the much-discussed role of Dutchbat, the Dutch government, and the UN, the lingering questions about guilt, responsibility, as well as the recognition of suffering. One dimensional narratives on the genocide are deeply entrenched and politicized in both Bosnia and the Netherlands, making it challenging to find room to empathize with the stories that emerge from both side. Historically, the relationship between veterans and survivors is shaped by questions of power, privilege, and institutional interferences, making it hard to reconstruct a free space that is so necessary for open conversations.

Reaching equality in encounters implies an ‘unlearning’ of earlier acquired perspectives, narratives, and worldviews (Everingham et al., 2021). As such, the success of an encounter is dependent on the willingness of visitors and hosts to think and to do differently. This might be challenging in a context in which visitors and hosts have diverging memories and interpretations of a conflict, as well as different expectations and needs for the encounters themselves. And yet, with a careful design and implementation, encounters with ‘the other’ could have a transformative potential (Soulard et al., 2021), and the possibility of improving the well-being of the participants. Therefore, we ask: what are the conditions needed to enable meaningful and transformative encounters with ‘the other’ in a post-war context? We analyse the experiences of Bosnian survivors, veterans, and their co-traveling family members all of whom are participating in the encounters in different ways. Our study is part of a larger research project that studies the entangled experiences of returning veterans. The analysis presented is built on ethnographic fieldwork conducted in cooperation with Bosnian women survivors and with returning veterans and their partners, in the form of group interviews with survivors as well as observations during multiple veteran return trips. Additional data has been collected with stakeholders in Bosnia and the Netherlands, through institutions such as the Potočari Memorial Centre, the Dutch embassy in Sarajevo and the Netherlands Veterans Institute.

Our ethnographic study adds, on multiple levels, to a body of existing research. While dialogue is a recurring concept in conflict resolution, our focus on the encounter allows us to take into account everything that happens outside of language too—particularly relevant in a setting in which participants do not share the same language and are dependent on interpreters. In these instances, both survivors and veterans suffer from an often one-dimensional framing of their respective group. Such framing not only reduces individuals to one specific aspect of their identity, but also ignores the significant individual differences that exist within those two groups. We argue that this framing might be the root of some of the tensions that emerge during the encounters, as polarizing the two sides tends to encourage an unproductive ‘us vs. them’ stance. For this reason, we propose a relational approach, in which we bring together the experiences of different groups of survivors, veterans, and their partners, allowing for a pluralization of identities and roles. Moreover, our study foregrounds institutionalized and politicized hierarchies present during the encounters (see for instance Cleven and Saul, 2021). Recently, researchers have scrutinized encounters between travellers and ‘the other’ (Pfoser and Keightley, 2021; Everingham et al., 2021), highlighting the power relations between travellers and local mnemonic communities. This approach acknowledges the inequalities between visitors and hosts—inequalities pertinent to interactions between visitors and local communities—as well as addressing the political and institutional dimensions that this form of visiting constitutes (Lisle, 2016). This is particularly relevant in a highly gendered post-war context in which the visitors are closely connected to military power, masculinity and implication (Rothberg, 2019), while the discourse on survivors revolves around victimhood, femininity and innocence (Helms, 2013). By taking into account the politics and power relations inherent to the practice of visiting and the enduring inequalities that the post-war context generates, we aim to understand the potential of encounters with ‘the other’ more fully.

To understand what qualifies the transformative potential of the encounters, we bring in theory from peace-building studies and conflict transformation. In particular, we refer to the work of Lederach’s (2005) on the ‘moral imagination’ as a strategy to ‘restory’ the past and future, and explore the conditions needed for such restorying. Encounters such as these are situated in cultural contexts, where past and present discourses about the Bosnian war impact on how memories and experiences are made sense of or/and spoken about. In so doing, we connect the experiences of the people studied to different discourses, narratives and national memory cultures revolving around the genocide and its aftermath.

This article starts with a discussion on dominant national narratives and collective memories of the genocide in Srebrenica—narratives that significantly influence how encounters are interpreted by participants and the institutions involved. These national narratives and collective memories have contributed to a narrowing of perspective and, at times, they have stood in the way of meaningful dialogue. By exploring these patterns, the article highlights the socio-cultural barriers that shape these encounters, offering insight into how narratives can be deconstructed to foster genuine understanding. The analysis is divided into three parts. The first two parts consist of a discussion around the existing discourses and narratives that emerge among the survivor and veteran communities, with an analysis of the impact of these narratives on the current encounters. The third part is about the observed interventions to these narratives, reflecting on their potential and also their pitfalls.

## Dominant perspectives on the Srebrenica genocide

In the almost three decades since the genocide, specific narratives and discourses have developed on ‘Srebrenica’, in particular within the Bosnian and Dutch national contexts where particular interpretations of and perspectives on the genocide and its aftermath have become dominant and widespread.^2^ Dominant narratives have a strong impact on how historical events are remembered, recounted and made sense of (for example Meretoja, 2023). Moreover, such narratives shape group identities, actions and politics (Liu and Hilton, 2005). Even though attempts have been made to nuance existing narratives, it is difficult for ‘other’ voices to be seen and heard, forcing veterans and survivors to take up opposing positions. As such, these dominant narratives obstruct chances for transforming the relationship between groups (for example Bilali and Mahmoud, 2017, p. 77).

### Dutch perspectives: from national failure to individual trauma

The Dutch public response to Dutchbat’s involvement in Bosnia initially emphasized the weak performance of its military (Uittenbogaard, 2024)—an image in line with dominant international perceptions of Dutchbat (Algra et al., 2007, pp. 403–4). Later, another narrative surfaced, in which the Bosnian war and genocide were seen in the context of the ‘impossible mission’ the Dutch government assigned to its military in the name of a UN operation. A mission with a limited mandate that focused on maintaining neutrality while keeping peace in Srebrenica—a mission doomed to fail, resulting in a national trauma (Rijsdijk, 2012). As such, nowadays, the Dutch collective memory of the genocide revolves around a story of military and political failure. Veterans and their representative institutions create a discourse that moves away from questions of guilt and implication and instead points to veterans as victims of political and international forces.^3^

As a result, the public debate revolves around a trauma discourse that emphasizes the consequences of the deployment for a specific group of veterans: those present during the fall of the Srebrenica enclave. This focus on trauma indeed reflects the fact that almost a third of the Dutchbat 3 veterans report to (have) experience(d) negative consequences associated with their deployment to Srebrenica (Olff and ARQ Nationaal Psychotrauma Centrum, 2020). Moreover, the majority of the veterans still feel they have missed a sense of reward and recognition (Olff and ARQ Nationaal Psychotrauma Centrum, 2020). However, and without the intention to diminish the suffering of individual veterans, such a focus on personal trauma in the Dutch national narrative has particular consequences. First, it creates a hierarchy in victimhood between different (groups of) veterans. Second, the dominant image of the ‘traumatized veteran’ limits veterans to position themselves differently (Driessen, 2021). Third, by shifting the attention from questions about (political) responsibility for violence to victimized individuals, a ‘social phenomenon is pathologized’ (Dawson, 2017, pp. 38–39). In this way, the dominance of the trauma discourse hampers politicians, military organizations and veterans from addressing their implication. Moreover, it disallows a multi-dimensional story of the Dutchbat 3’s deployment.

### Bosnian perspectives: gendered narratives of victimhood

In Bosnia, the consequences of the genocide are still felt daily. Survivors and relatives of victims, in particular those living in the Republika Srpska (RS), are confronted with a genocide denial propagated by the government of the RS, as well as with enduring ethno-nationalist propaganda and ongoing tensions. This political reality has shaped the current memory culture, in which the Potočari memorial and activist ‘mothers of Srebrenica’ fight for the recognition of the genocide by alluding to a discourse of victimhood. Central in this discourse is the search for historical evidence, justice, and documentation of the stories of victims and survivors. However, within this memory culture, some voices—those of the Potočari genocide memorial and the ‘mothers of Srebrenica’—seem to overshadow others, which has consequences for how wartime losses and violent experiences are made sense of by survivors and how they position themselves within this discourse of victimhood. The dominant focus on Srebrenica also means that there is less attention to other genocidal killings and ethno-nationalist violence that took place since the start of the Bosnian war (Jacobs, 2017).

In her work on Bosnian women war survivors, gender and victimhood (2013), Elissa Helms argues that allusions to Bosnian/k women victimhood are ambiguous, because they are subject to reinforcing conservative gender norms, ethnic stereotypes and orientalist representations (p. 6–9). Such allusions can be very powerful, resulting in gendered narratives of victimhood imbued with references to motherhood, innocence and moral purity that are easily accepted in public and political spheres (p. 4). This can be harmful, as these narratives feed into a portrayal of womanhood that is passive and domestic, thereby hindering women to take up different, more active roles (Smith, 2016), or to speak about topics like sexual violence and rape that do not align with ideas of virtue and purity (Jacobs, 2017, p. 434). Moreover, these allusions can be used to promote an ethno-nationalist discourse that does not allow for much nuance, nor does it do justice to the complexities of (post)war experiences. As such, and although there are exceptions (see Helms, 2010), if they aim to become more active, women survivors seem bound to specific gendered and politicized narratives of victimhood.

### Earlier encounters between survivors and veterans

Both veterans and survivors construct their stories in relation to the hegemonic narratives circulating about what happened in ‘Srebrenica.’ Impacted by these narratives, the relation between survivors and veterans remains tense. Survivors feel neglected by the UN, national, and international communities, who they felt had not cared to protect them. Survivors do not feel supported by the Dutch state or by the Dutch veterans. The decision of the Dutch government Dutchbat soldiers in 2006 to recognize and decorate the soldiers for their efforts in dire circumstances, received criticism among Bosnian survivors and the Dutch-Bosnian community (Van den Berg, 2014). Feelings of anger and distrust remain amongst survivors and questions linger about the willingness of the Dutch government, the military organization, and veterans, to empathize with Bosniak perspectives.

Organized encounters between survivors and veterans have been taking place on a small scale. For instance, there have been trips and encounters initiated by the Dutch peace organization PAX and the Camp Westerbork Memorial Centre in 2007 (Van den Berg, 2014). Reports of these meetings are not positive. These encounters have been described as difficult and confrontational. This might partly be caused by the presence of the international press and the way these encounters were setup where there were only a limited number of veterans who were placed opposite a large group of survivors who were firing questions veterans felt they could not respond to. The more informal aspects of these encounters seem to have had a better outcome (Van den Berg, 2014). The negative experiences of these encounters between survivors and veterans have been shared widely within the Dutchbat community and they continue to shape their perception of the survivors. As such, at least until now, the current encounters between survivors and veterans take place in a context where perspectives on the genocide and its aftermath, in particular concerning often gendered questions about guilt, implication, trauma, innocence and victimhood, lack the necessary space that is needed to have meaningful engagement with each other. The question then is, what is needed to encourage mutual listening in order to open up spaces for new perspectives where there is recognition of individual experiences?

## The moral imagination: restorying narratives of the past and present?

In his well-known work on peacebuilding, Lederach (2005) explores the conditions needed to transform existing patterns and cycles of hostility and violence. His focus on philosophy of life, individual meaning making and humanization opens up a space for discussion around the structures that underlie processes of conflict transformation—a focus that is often missing in more practical and instrumental work on conflict and reconciliation.

Lederach refers to the moral imagination as a means of building peace by reshaping existing narratives on the past and present. Building moral imagination relies on four different practices:

“The moral imagination requires the capacity to imagine ourselves in a web of relationships that includes our enemies; the ability to sustain a paradoxical curiosity that embraces complexity without reliance on dualistic polarity; the fundamental belief in and pursuit of the creative act; and the acceptance of the inherent risk of stepping into the mystery of the unknown that lies beyond the far too familiar landscape of violence.” (p. 5).

For Lederach (2005), to facilitate change, the moral imagination demands us to think—with curiosity—about the roots of violence and its impact on us, while also allowing ourselves to explore the creative process needed to imagine the past and future differently.

Narratives take up a central role in the functioning of the moral imagination. As narratives are closely bound to identity formation, both on individual and group levels, they have a profound impact on how people define who they are and what their past entails (2005, p. 142). Traumatic events occupy an important position in these narratives: a selective series of events is often told and retold through generations, thereby continuing to shape group identities. However, such events are also all too often used as arguments for revenge or defence.

According to Lederach, people must explore and understand the narratives that are so foundational for who they have become. However, in cases of conflict, violence, or repression, these narratives could be regarded as broken. Finding and reformulating the narratives that capture lived experiences, identities and histories lost within a community constitutes a way forward to healing. In this way, peacebuilding comprises a ‘restoration of narratives’ (2005, p. 146). Lederach calls this ‘restorying:’ “Restorying as imaginative narrative looks for the deeper social story and meaning, not just of what happened, but how stories are connected to a far more profound journey of discovering what these events mean for who we are as both local and global communities” (2005, p. 147). Such a restorying of the past gives meaning to life and social relationships on a profound level.

Relational thinking is at the heart of Lederach’s approach: by considering ourselves and our stories to others, to the past and the present, and by creating platforms that help to build unusual relationships and interactions, we can avoid binary thinking and address the complexity that relationships entail. Conflict and violence tend to fall back on binary oppositions; relational thinking makes it possible to transgress such oppositions. However, moving beyond those oppositions means being willing to take a risk: to leave the safety of what is known and “seek constructive engagement with those people and things we least understand and most fear” (2005, p. 173). Only then, can cycles and patterns of violence be broken.

Lederach’s work on the moral imagination presents important insights that could be of use when attempting to ‘restory’ narratives on Srebrenica and its impact on survivors and veterans. Concretely, we can ask: what do mnemonic communities need to imagine the past and future differently? Some examples might be found in recent attempts to nuance existing narratives, for instance through creative projects in which veterans and survivors meet or in which ‘alternative’ stories are told, such as theatre plays or art exhibitions that have recently been initiated in the Netherlands.^4^ Encounters between survivors and veterans might be seen as an attempt to incite the moral imagination, and develop a moral sensitivity to understand and feel the perspectives of others (see also Johnson, 2016). In this way, restorying the past might help to envision a different image of the future, a future in which narrow perspectives on identity (survivor, veteran) could be broadened and refined. In this way, restorying one-dimensional narratives on Srebrenica might help survivors and veterans create a different image of themselves and each other, and, in the process healing becomes possible.

In practice this might be easier said than done. Dominant narratives hold a firm grasp over people and communities. They are hard to refute—it is always easier to fall back to what is known and believed than to move into unknown and inconvenient place (Lindemann, 2020). Can the addition of alternative perspectives bring about change to dominant perspectives? Are these different perspectives merely co-existing without interacting with each other? Are the existing hierarchies so entrenched that they remain intact? Lastly, although encounters with the ‘inconvenient other’ (Berlant, 2022) could theoretically be regarded as productive, this does mean creating a safe space that is not harmful to people, but that is enriching and conducive to overall well-being.

## Method

### Ethnography

This study is based on ethnographic fieldwork conducted in Bosnia and the Netherlands in 2023. During this empirical study, we specifically focused on the expectations, experiences and evaluations occurring between survivors and veterans (Vermetten et al., 2024), as well as on the role of veterans’ partners (Driessen et al., 2024). Because we could interact with our participants over a longer period,^5^ we were able to analyse gradual developments and changes in attitudes towards these encounters that are often missed when the research is based on single trips and intermittent encounters.

### Participants

The encounters between survivors, veterans and their relatives are organized by the Netherlands Veterans Institute, the Potočari Memorial Centre and Snaga Žene, and take place on the instructions of the Dutch Ministry of Defence. Thus, there is an institutional dimension to the encounters, with the possibility of unintended consequences for the participants. Intermediaries, such as institutions, could have a ‘neutralizing’ effect on the encounters because of their intrinsic avoidance of everything that can cause trouble (e.g., Pfoser and Keightley, 2021). This could also inhibit ‘risky spaces’ that are needed for transforming particular interpretations of collective memories (Lederach, 2005). As such, it is important to include the institutional context when studying return trips, as this institutional dimension may be impacting on the course of the encounters between survivors and veterans.

### Dutchbat 3 return trips

In 2020, the Dutch government allocated sufficient funding for return trips to Srebrenica for Dutchbat 3 veterans. The trips are part of a larger series of Dutch governmental gestures to enhance veterans’ rehabilitation and recognition (Borstlap, 2020). From 2023 onwards, approximately 850 veterans and their partners, friends or relatives have had the opportunity to return to Bosnia in groups of approximately 10 participants. The 5-day trips include site visits. These site visits were impactful to them during the deployment. The group also participated in touristic visits, for instance to Sarajevo. Meetings with women survivors are a recurring part of the return trips. Participating in the meetings is not obligatory, and in some cases, veterans decide not to join. The meetings are guided by co-traveling military chaplains, as well as employees of Snaga Žene and the Potočari Memorial Center, who also provide translations to the participants.^6^

### Snaga Žene

Snaga Žene is a women’s association founded in Tuzla in 1999, which supports women survivors of war and sexual violence in different ways: from economic and legal support to health and psychological care (Antić-Štauber, 2017). Snaga Žene hosts different women groups in Srebrenica, Potočari and Tuzla, who meet every other week. In Tuzla, Snaga Žene has created a garden where the women plant, harvest and sell herbs and flowers as part of their economic rehabilitation and as occupational therapy. The veterans and their partners visit this garden during their trips and meet and speak with the women working there.

### ‘Mothers of Srebrenica’

The ‘mothers of Srebrenica,’ located in Sarajevo, have become the faces of survivors of the genocide (e.g., Jacobs, 2017, p. 426). The ‘mothers’ consist of various associations of women survivor groups, representing different regions or victim-groups. The ‘mothers’ focus on collecting evidence of the genocide and seeking justice, for instance through the court case that they filed against the Dutch state. As such, and in stark contrast to the women of Snaga Žene, the ‘mothers’ have taken up a political position and they have come to embody the voice of the genocide survivors, advocating for their case all over the world. During their return trip, veterans meet the ‘mothers’ at the Potočari memorial, participate in a small memorial ceremony, and engage in conversations.

### Material included in/from the fieldwork

In total, we had conversations with 27 women survivors, 16 return trip participants, 8 guides, chaplains and social workers, 8 stakeholders, were participant observers in 3 sessions with veterans and survivors, and had many more smaller conversations with people involved in the encounters from both sides. Translation has played a major role in what we were able to ask and discuss with the survivors—however, by recording and translating group interviews that predominantly took place in Bosnian, we have tried to capture parts of what was lost in conversation. Fifteen hours of interviews have been recorded, transcribed and, where needed, translated. When recording was not practical or appropriate during the trips, extensive fieldnotes were taken throughout the day, and these notes were compared and discussed amongst the authors. All participants consented to take part in the research. The project was approved by the Ethical Review Committee of the University of Humanistic Studies.

### Narrative analysis

Narratives function as mediators between the individual and the collective—they allow us to narrate our experiences in understandable and transferable ways, thereby shaping what we can tell others (and ourselves) (Bruner, 1987). As such, narratives give insight into dominant cultural and political conventions and reveal how people make sense of the world. In contexts where such conventions seem to overpower the recounting of other stories and experiences, it is relevant to study these narratives in-depth. Doing a narrative analysis requires extensive knowledge of the context in which these narratives are performed (Meretoja, 2023). Long-term ethnographic research is conducive for developing such a sensitivity.

Our fieldnotes and transcripts have been analyzed thematically by concentrating on narratives about the genocide and the Dutch deployment to Srebrenica and interventions to them, attitudes of survivors and veterans towards each other, as well as expectations, experiences and evaluations of the encounters. The data has been coded inductively and organised into broader descriptive categories, using a manual coding strategy. All categories and codes relevant to our study have then been connected to the narratives pertaining to them. For instance, the code ‘not our story’ belongs to category ‘attitudes towards the other’, which we then related to the broader Dutch narrative on Bosnian ‘others’. In the analysis, we specifically focused on the performance of dominant narratives as well as moments of interventions to these narratives. Data on the veterans/partners and survivors has been analysed separately, in order to maintain their specific vocabularies, expressions and perspectives, before being connected in the analysis below.

### Researchers

The research was conducted by four researchers with a Dutch or German background, living in the Netherlands. The fieldwork included three trips to Bosnia: one in which we visited the women of Snaga Žene, the ‘mothers of Srebrenica,’ and various stakeholders. While Siri, Nicole and Jeannette conducted the group interviews with the survivors, allowing for an all-women conversation, Eric interviewed the stakeholders such as the Potočari genocide memorial and the Dutch ambassy in Sarajevo. Furthermore, Siri fully participated in two government-organized veteran return trips to Bosnia, during which veterans and their partners took part in organized meetings with women survivors, also attending preparation and evaluation meetings to do with these trips in the Netherlands.

The four authors had different roles throughout the project. Siri had the lead in the data-collection, analysis and writing, partially relying on earlier work on veteran travel (e.g., Driessen, 2021; Driessen et al., 2024). Nicole and Jeannette gave feedback throughout the project and provided extensive comments to drafts of the text; Jeannette from her perspective as a researcher and military chaplain, and Nicole from her expertise in memory studies and transformative justice. Eric initiated a funding application for the project, provided access to the field, and shared insights from his perspective as a military psychiatrist.

Doing ethnographic research, in particular when it takes place over a longer period of time, relies on connections and collaborations with individuals and institutions. These encounters can also lead to specific expectations of the research as well as attitudes towards us and them. Our position as researchers representing the Netherlands, our gender identity, and Eric’s affiliation with the Dutch military, including his consultations on return trips, may have impacted on what we have seen, heard, and emphasized. For instance, the first, second and third authors’ female identity will have sensitized us to questions pertaining to gender relations, the performances of military (hyper) masculinity and power imbalances (e.g., Baker et al., 2016), and—to some extent—attuned us to the experiences of partners and survivors. This also meant that our position towards the predominantly male veterans might have been biased and where we felt less attuned and more an outsider.^7^ Our own familiarity with Dutch narratives and Dutch attitudes towards ‘Srebrenica’ may also have resulted in a heightened awareness of these narratives and attitudes.

## Analysis

Discourses about war, about genocide and about victimhood are likely to impact on the way both Dutch veterans and Srebrenica survivors talk about their experiences and about each other. The prominence of these mostly one dimensional discourses has ramifications and cloud the way survivors and veterans interact. Thus, it is important to question those discourses. Although it might be disturbing to problematize something familiar, in the analysis below we argue that such questioning offers a way forward for developing more sensitivity towards the other’s position. Our research suggests that interventions interrupted acquired perspectives and attitudes and thus created space for a different kind of conversation. We also saw how precarious it is to do so, and that, even after these interventions, participants tend to fall back to old and more familiar perceptions of each other. In order to fully understand the dynamics taking place in these encounters, we first discuss the dominant discourses present among the participants, starting with the veterans and followed by the survivors. Then, we move to discussing the interventions made during the encounters and we evaluate the potential of these ‘new’ encounters as well as the pitfalls when attempting to ‘restory’ the past. Each section starts with an example of a particular perspective, interaction or attitude.

### Do not blame us, hear us: Veteran’s perspectives

With a group of returning veterans, we visited the memorial exhibition in Potočari, located at the former UN compound of the Dutchbat military. Traces of their presence are still visible, for instance in the form of graffiti Dutch soldiers drew on the walls of their quarters. However, after visiting, a feeling of disappointment emerged among the veterans, and some of them seemed to have hardened their perspective. They experienced the exhibition as ‘one-sided’, call it ‘the Muslim story’, and share this feeling in the group. They are disappointed: the memorial shows little of ‘their story’. They expected to see more of Dutchbat’s history at the site, but cannot find it. For instance, there see no mentioning of the powerlessness they experienced. Moreover, some veterans find it difficult that Potočari has changed so much since 1995; that what once served as their compound, is now being renovated, adapted, plastered over. A fear that they will not be able to tell their side of the story emerges.

Many of the participating veterans regard the return trip as a means to close off a difficult period in their past, to find support in the group and to ease feelings of guilt. They come to Bosnia in search of relief. For instance, a veteran mentioned to participate because he sought to “*work through the deployment and close it off*.” Another wanted to “*add a new chapter to the book Srebrenica.*” Institutionally, the trips are framed as a governmental gesture designed to enhance veterans’ rehabilitation and recognition (Borstlap, 2020). As such, return trips can be seen as an attempt to compensate veterans for the ‘impossible mission’ they were sent on and their consequent suffering. The Netherlands Veterans Institute, concerned with the implementation of the trips, regards the return trip as a moment to ‘make new memories’ and thus, to add positive images to mostly negative ones of 1995 (nlveteraneninstituut.nl). This brings with it an understanding of the return trip as a means to offer veterans relief whether this be in the form of recognition, compensation, or/and personal well-being.

In this context, the purpose of the encounters between veterans and survivors during return trips remains somewhat undefined. Both veterans and organizers have a predisposed resistance to the encounters. Some participants mentioned to “*prefer to spend their time differently*,” while others decided not to join the meetings with the ‘mothers of Srebrenica’ at all. Questions about the goal of the meetings lingered among participants. Why are these meetings being organized? Who is actually benefitting from them? How could the encounters contribute to the recognition, compensation or wellbeing of veterans? These hesitations felt by the participants suggest that they regard meeting the survivors as something threatening and dangerous to them. For instance, a veteran mentioned several times that he “*hoped not to be made to feel guilty.*” He feared that participating in the encounter would turn out badly for the veterans and that he might experience quite the opposite of what was being promised with the return trip. The encounters are often seen to be risky—both at the level of the individual and for the institution.

To understand veterans’ and organizers’ hesitations about the encounters with survivors, it is helpful to explain Dutchbat’s preparation for the deployment to Bosnia. When veterans speak about Bosnia, prior attitudes about the country and its citizens resurface. These attitudes can be traced back to the information that veterans had received in preparation of the deployment—drawing an image of the western Balkan as a region of perpetual ethnic conflict, a place of risk, where situations escalate quickly and violently. This resulted in an ‘othering’ of the region and its inhabitants, an othering strengthened by the UN mandate in which soldiers were not allowed to be in contact with local populations. This was to ensure that they keep their ‘neutral’ position and that they do not become acquainted with the personal experiences of citizens. This othering has persisted among some of the participants after the war and it has been reinforced by the negative reception of Dutchbat—references to perpetration and accusation—tensions between survivors and veterans and a growing societal anti-Muslim sentiment. This can stand in the way of seeing the conflict in a different perspective than the one they had got so used to over the decades and it limits their capability to think beyond ‘us vs. them.’ It also impacts on their outlook on the encounters with survivors.

A well-known conviction felt among veterans and their representatives revolves around the feeling that they have been ‘used’ by international politics and the UN for purposes they were not aware of at the time. As a consequence, they are wary of outsiders and the tendency to rely on them. This wariness carries over into their return trip. At the institution level there is also the allusion to risk and danger that surfaces in the way the encounters are spoken about. Why bring ‘our’ veterans into danger by confronting them with survivors? Someone warns us: “*Before you know it, the veterans are again another instrument in a larger story, another position over which they have no influence.*”

When speaking about the desired outcome of the encounters, some veterans emphasized their need to experience support from the survivors. “*It would be nice if there would be a kind of ‘thank you for coming here’ from the population, that’s what I need.*” Often, this search for support clashed with images of Bosnia/ns as a place of fear, risk and danger. In one of the encounters, a veteran told the group that he found it hard to speak about the emotions relating to his deployment and that he had silenced himself for a long time. A survivor encouraged him to start talking, as this had helped her in her process. “*Think about what you do have*,” she said, “*you are still alive, while my husband was killed*.” The veteran experienced her remark as a critique, a warning that he should not complain too much and this resulted in him reflecting negatively on the encounter.

The example indicates how an initial attempt to search for an understanding and support is usurped by the fear of not being given the space to tell a story. Other veterans also struggled with this relationality: “*The pitfall is that we are there for them instead of the other way around.*” These veterans appointed the tensions between the promise of the return trip as means for improving veterans’ wellbeing or promoting recognition, and the role-reversal during the encounters, where the stories of the survivors are experienced as overpowering those of the veterans. Still, once a veteran felt that their quest for support was acknowledged, there was visible relief, for instance when a ‘mother’ stated that “*we are not speaking bad of you, because we know you got orders*.” Her remark gave the participants the impression that other stories could now be told. Moreover, after the encounter with the ‘mothers,’ a veteran said that he did not understand what all the initial fear and hesitations had been about and that he experienced the meeting as being pleasant. As such, to some extent, the meeting seemed to allow for mitigating earlier-acquired apprehensions.

Most veterans embark on a return trip for personal gains: to be relieved of the past, to find closure. Veterans express a need to be heard and supported—they long for a more nuanced understanding of what had taken place and for empathy. Yet, the logic of seeing the encounters with survivors during the return trip as a gesture of compensation and recognition, leads veterans to feel hesitant about the purpose of the encounters: why are difficult and confrontational encounters part of a trip that is organized to compensate veterans for their suffering? This hesitation is fuelled not only by earlier attitudes that existed towards ‘the other,’ but also by the feelings of fear, risk and danger that dominate their discourse, both on an individual and institutional level—a discourse that has its roots in attitudes towards the Bosnian/k ‘other’ that developed during the UN mission. Moreover, fears of being side-lined, of not being able to tell their story and be listened to, and of being accused of perpetration, make veterans and organizers wary to involve in the meetings. And although some of the veterans look back positively on the encounters, the way prior attitudes and the discourses that go with them hangs over the encounters is something that stood in the way of meaningful conversations.

### Witness, testify, and speak out! Bosnian survivors’ perspectives

We are in a small living room in the city of Srebrenica. Although it is snowing outside, the room is packed with women of different ages. Some of them have been participating in these meetings for years already, others joined much more recently—for one participant, this is her first meeting. Food is being prepared while the women update each other on their families, their health, politics and international affairs. The Director of Snaga Žene takes the word, and introduces us, researchers from the Netherlands who are interested in their histories, in particular their experiences of the meetings they have had with Dutch veterans. The women welcome us, and immediately reply with a question: “*Why did the Dutch soldiers give their uniforms to the Serbs to wear and walk among us?*” The question about this specific memory turned out to be a recurring one, posed in all the conversations we have had with survivors. In due course, we realized that the question is more than just a request for information: it is an appeal to convey some of their experiences of war and genocide to us, experiences that pertain to existential emotions revolving around being betrayed, neglected, not being taken care of, dehumanized.

When speaking with the women survivors about their experiences with Dutchbat veterans, the discourse revolves around the soldiers witnessing unfolding crimes. “*They saw more than I could,*” told a survivor, emphasising the relative freedom the Dutchbat soldiers had to move around. References to Dutch soldiers as witnesses to Bosnian-Serb crimes are a key element in the women’s stories—in their eyes the soldiers occupied a position of power and access to information they themselves did not have. These references to power result in veterans being seen complicit in the crimes. For instance, a woman in Tuzla explained: “*I was in Potočari when those Dutch soldiers were there. Unfortunately, they separated my father from me. In front of the Dutch soldiers.*” Her observation has an emotional undertone: the pain of losing her father, who was not seen to be worth saving alongside the false sense of protection offered by the UN.

In the survivors’ accounts of their interactions with Dutchbat soldiers, questions about specific memories are often interwoven with more existential questions. Hana,^8^ who survived the war as a young girl, told how she needed to search for food on trash piles created by Dutchbat soldiers, and the humiliation she felt because of it:

“And the soldiers, as soldiers I hold it against them because they could have sometimes given something [ie food] that was packaged. When we came to the trash heap, they could have given us (?) for the bags but sometimes they would take it and they would throw it. As far as they can, they throw it so you would have to go get it, you know.”

Having to go to the dumping grounds to search for food for her parents and siblings was a humiliating experience for Hana, and it recurs in her story multiple times. The specific lack of care—not giving her the food, but having her collect it on the thrash pile, aggravated feelings of dehumanization that she connects to the presence of Dutchbat and also to a more general sense of neglect. There is also a cultural aspect to the humiliation she experienced: “*I do not know why it was so, did they hate us Muslims, Bosniaks so much?*” The survivors raised doubts such as these several times. In this way, they tried to convey their painful memories and stories—such as the one about the switched uniforms or having to visit the trash pile—that revolve around feelings of dehumanization.

Survivors appoint the privileged position of Dutchbat soldiers. Soldiers were not a target, while the Bosniaks were: “*They shot at everyone, projectiles, but they did not shoot at UNPROFOR*.” These feelings relate to the betrayal felt after the enclave was attacked: “*It was during the war, when they came…my Amer and I, as far as the two of us were concerned, we trusted [them], we were so sure that nothing could happen to us because they were there*.” Realizing that the promised UN protection against Bosnian-Serb troops was not going to take place gives rise to existential questions about the reasons why it did not: “*They slaughtered my mother-in-law, they killed [unintelligible] killed all the people…they enslaved me, me and the children…and why?*”

Allusions to Dutchbat soldiers’ unique and privileged position as eyewitnesses are repeated over and over again during the survivors’ quests for testimony and truth-telling. This is predominant in the more political discourse of the ‘mothers of Srebrenica’. When asked about her opinion on the visits of Dutchbat veterans, one of the ‘mothers’ explains:

“It’s nice when they come, it’s nice when we hang out together, but the most important thing is that they say what they have seen, what they have experienced, what they have survived and that it is noticed. So that it is not forgotten, so that it does not happen again. We thought never again, but as we talk Ukraine is burning. Mothers cry.”

The mother’s concern is about the lack of engagement from veterans that they have felt until now. Impacted by the past contentions, the cases of the veterans and the survivors have been taking place in isolation. As such, the discourse can be read as a plea for support from the veterans, in which survivors, as main victims of the genocide, set out the conditions for these visits:

“I said it today, this morning, today…if they come here to have their pictures taken, I suppose, to pay their respects, and not to speak out then we have nothing to expect from them and they need not come.”

“If their visit is only to heal themselves and we do not feel any support or apology for that, then that is something that makes no sense.”

In their appeal for support, the ‘mothers’ emphasize the importance of long-term encounters. Veterans cannot simply come as tourists or visitors, who take a snapshot and leave: they have to be willing to invest in the relationship if the encounters are to be successful. It is possible to read into the survivors’ appeal that there is the potential for a space to open up, showing that there is some form of relational thinking—testing the willingness of veterans to engage with the needs of the other, as well as to make a commitment to recognize existing hierarchies of victimhood.

The survivors’ emphasis on witnessing, testifying and speaking out echoes other discourses that have emerged from other wars and genocides, where the focus is on collecting evidence, on truth-telling and on finding justice. It is through this witnessing, testifying and speaking out that survivors are able to connect their cause to other historical searches for justice. However, the question is whether a continuing focus on testifying and speaking out is the most fruitful way when wanting to make a connection between both groups? Individual veterans do not have the answers to questions that are actually meant for military leaders and politicians that were in command—yet, as ‘representatives’ of the Dutch state, veterans are often expected to be able to provide these answers. This is unfortunate because the veterans feel that the encounters are unequal and heavily politicised, which discourages them from wanting to participate fully in the encounter. Still, in instances where veterans were able to give precise answers to questions about what they had seen during the fall of the enclave, this helped the conversation forward—they were taken seriously and were seen to be honest. Listening to the survivors’ wartime experiences with Dutchbat soldiers and their questions to the veterans about providing evidence and speaking out was the precondition for a meaningful encounter to take place. Veterans had to listen first before other topics could be addressed; showing a willingness to engage with the survivors’ perspectives. Listening to each other stories is a way to connect. In the words of Emina, a long-time member of Snaga Žene Tuzla:

“Because you know what it means when I [speak] directly to someone who was there of those soldiers, directly looking in his eyes tell him what I survived, me personally. And he says his side. And that we try like that (…) [This] should be [done] for the unburdening of us and them.”

The ‘mothers of Srebrenica’ and the women of Snaga Žene have a common discourse, in Emina’s words we can recognize the framing offered by Snaga Žene, that suggests a future-oriented and relational dimension. Both groups need each other to heal. Emina continued to highlight the outcome of the encounters:

“And [let us] try to tell the real truth to one another, maybe that way it would be a little, a little easier for us, well compared to the pain that we survived it can’t be much [easier], but that we come to some kind of goal, that we’re not saying they could have, they couldn’t have, this and that…that instead we go to the end, to the end goal.”

Emina is emphasizing, even though perhaps only implicitly, the need for survivors and veterans to come to terms with each other—and she is also suggesting a future in which the relationship between survivors and veterans could evolve. Emina also suggests that for herself, questioning and blaming each other is not a constructive way forward. For her, the ‘real truth’ might rather be about shared feelings of humiliation and dehumanization, about being seen and being treated humanely.

### Restorying past and present: Searching for interventions in solidified discourses

Another meeting at the Netherlands Veterans Institute, almost 2 years after the start of the return trips. More than 200 veterans and partners have visited Bosnia by now, and met the genocide survivors. This time, not only the Dutch organizers are present, but also Bosnian representatives, including the ‘mothers of Srebrenica’, as well as (former) Dutch politicians, ambassadors and other governmental persons. Different people get the opportunity to talk and reflect on the encounters that have taken place between survivors and veterans. A veteran takes the floor. He appreciates the survivors for visiting the Netherlands, thanks them for their hospitality in Bosnia, mentions how he obtained more insight in their stories and their suffering, and tells how he hopes to strengthen the relation with them. The veteran’s remark stands out: while most contributors seemed absorbed by their own story, this veteran made an effort to think relationally.

The previous sections analysed the dominant frames and discourses present among veterans and survivors. Despite the omnipresence of these frames and discourses, there were moments in which successful interventions were made where it was possible to confront predetermined discourse. These interventions had different formats. Below, we will discuss how these interventions might be seen both as an attempt to restory past and present: interventions by veterans’ partners, connecting through the creation of shared discourses, and also a pitfall: falling back to old narratives.

#### Interventions by veterans’ partners

When offered the opportunity to return to Bosnia, veterans were explicitly invited not to go alone but to bring a ‘relation,’ in most cases a partner. Currently, organizers offer different kinds of trips: with and without relation, causing women survivors to meet veteran-only groups as well as mixed groups. Undoubtedly, traveling in company of partners impacts on the return trip as a whole,^9^ and partners significantly influence the way that encounters take place between veterans and survivors.

During our final trip, the veterans were tense about the encounter with the ‘mothers of Srebrenica.’ While the majority of the veterans were nervous, some decided not to join the meeting at all. Once everyone was seated, one of the partners decided to break the tension and to start the encounter with a question, saying to the survivors “*we are so curious to hear your stories.*” This is an open invitation to the ‘mothers’ to tell their stories and an indication of the participants’ willingness to listen. The survivors spoke for a long time, while partners and veterans empathized, remaining silent and also showing support through bodily gestures. The partner’s opening question resonated well with the survivors, and it was a good starting point for a conversation, ending with an expression of a shared suffering. In this way, through the act of listening, participants could ‘bestow recognition on survivors and their suffering (Gobodo-Madikizela, 2016, p. 124).

The example illustrates how adding a different voice to the encounter—that of a partner—can change the course of the conversation. As civilian outsiders to ‘Srebrenica’—they had an intimate knowledge of the events and the impact of war affecting their daily life, and even though there was no direct involvement in the events, partners can provide a bridge between survivors and veterans. As such, they are able to intrude or/and break up the dualistic setting, allowing for the encounter to have more than simply two main narratives. This results in an “interaction with reality that respects complexity and refuses to fall into forced containers of dualism and either-or categories,” and as such inciting the moral imagination (Lederach, 2005, pp. 35–36). Gender plays a significant role. All co-traveling partners were women and thus there was a shared womanhood, sometimes motherhood, that created a connection between partners and survivors. The shared gender identity also meant that partners had the opportunity to support the survivors physically when the conversation became emotional, for instance by hugging or holding hands—significant gestures when you do not share a language. The presence of women partners provided an opportunity to neutralise or change dialogues that could otherwise have remained stiff and difficult. The ways the encounters were set up also meant that partners could take an active role in the return trip—and allowed them to reverse the classic ‘homefront’ setting, in which partners are predominantly seen as supporters and caregivers of the veteran (see Driessen et al., 2024).

In this way, by beginning the conversation and then carrying out much of the conversation, partners helped the veterans to engage in the encounter in a less direct or oppositional way. Moreover, because of the presence of the partners, veterans were seen not only as former UN soldiers. Through the stories of the partners, they also fulfilled the role of husband or father, thereby suggesting a far more nuanced identity than that of being just a military veteran. That partners could take up an active role in the conversation also meant that they had more agency as co-creators of the encounters—a sense of agency that is often lacking in the design of the return trip that focusses so heavily on the veteran. On the other hand, this also means that partners are burdened with a responsibility they might not have anticipated or desired (Driessen et al., 2024).

#### Connecting through the creation of shared discourses

Talking to veterans and asking questions helped survivors to adjust their image of the power that Dutchbat soldiers had during their presence in Srebrenica. In the words of one survivor: “*I learned that they in fact did not have any authority. According to their story.*” By gaining first-hand knowledge of the role of Dutchbat, this survivor could add a new image to those she already had of the veterans: an image that captures feelings of powerlessness that are so central in the veterans’ stories, a feeling also present in the accounts of the survivors. In this way, survivors connect with veterans’ stories by emphasizing shared feelings and experiences of powerlessness.

Allusions to a shared narrative of victimhood are also explicit in the institutional setting. Within Snaga Žene, survivors are encouraged to adopt a discourse of shared victimhood. When the Director proposed the encounters with veterans to a group of women survivors, she stated that:

“If you ask me this is a unique opportunity in the world. That those who are labelled as culprits, the soldiers, and you who are clearly the victims get together. It’s very rare that we have the chance to have those two groups meet. And that’s what’s happening right now. Because unfortunately, they aren’t the culprits but are also victims.”

Appointing similarities in the stories of both survivors and veterans is used as a way to connect. The words of Snaga Žene’s Director resonate in this extract between the Director and a survivor:

D: “…because look, it’s very important that they also hear, that they hear your stories and your questions why, and that night how it was, yeah, and all of that. Because look, they also aren’t at peace because this is not meaningless. They also have problems.”

S: “Maybe they do not sleep at night either.”

D: “That’s right, just like that.”

Constructing a shared narrative of victimhood, revolving around the experiences of 1995, feelings of powerlessness, living with PTSD, and feelings of (survivors’) guilt, appears to be a successful strategy: veterans felt seen as co-sufferers of the genocide, while survivors were able to add a new layer to their image of the veterans, that initially focused on traits associated with stereotypical military masculinity. Knowing that veterans also suffer(ed) helped survivors themselves feel recognized. Moreover, as we saw earlier, it allowed them to feel supportive towards the veterans based on their own experiences in dealing with trauma. In this way interactions between veterans and survivors assisted in restorying well-known discourses. What felt as purposeful neglect and dehumanization—thus as othering—is turned into a more meaningful truth, namely that the Dutchbat veterans were relatively powerless and following orders.

Nevertheless, although helpful, this kind of restorying also has its ambivalence as it moves away from questions of responsibility and guilt, that are uppermost in the minds of survivors, in particular the ‘mothers of Srebrenica.’ When these questions of guilt and responsibility came to the surface, it was difficult to dialogue. In these conversations, another narrative strategy seemed to be more successful: namely, to adopt each other’s discourse. This adopting was done in both directions: survivors confirmed veteran’s quest to be seen more fully: “*I know that Dutch veterans suffer from stigma. That is also why it is important to tell.*” In turn veterans repeated survivors’ pleas for testifying, for speaking out, and never to forget—refrains which resonated well and strengthened the idea of the collective mission to make sure that the memory of the genocide did not become one of denial.

Clearly, the creation of a shared discourse allows survivors and veterans to connect. Recognizing each other’s experiences and (political) missions, makes it possible to move away from one-dimensional interpretations of each other’s history and identity, and to create a more nuanced and shared narrative (Lederach, 2005, pp. 35–6). Is the shared victimhood a necessary condition to enable dialogue between survivors and veterans before other questions about responsibility can be answered, or does it ultimately hinder the desired connections? Meetings go well as long as there are acceptable discourses, but discussing inconvenient topics remains more troublesome. We see here the limitation of one-time encounters: working out these questions requires a much longer engagement between the groups. “Sustaining peaceful transformation in settings of deep-rooted violence requires a long-term view that focuses as much on the people in the setting of conflict building durable and flexible processes as it does on specific solutions” (Lederach, 2005, p. 47).

#### Falling back on well-known narratives

Despite their very real initial hesitation, most of the participating veterans reflected positively on the meetings. They felt relieved and experienced the meetings as much less stressful than expected: “*I think it is special that the population takes this attitude, I did not expect that. I think that if you sit opposite each other, with the population, without talking about the incidents, you can very well talk about sadness and loss*.” Another veteran, who did not join the encounter with the ‘mothers’, reported later to have a “*different view of the women*,” and that he “*also got what I wanted to see,*” after visiting Snaga Žene. Most veterans were happy with the meetings and experienced them as less of a burden than they had expected. Listening to the stories of the survivors gave them a sense of purpose: their willingness to listen and to empathize brought on the feeling that they could be instrumental in giving the much-needed help to the survivors.

However, despite their willingness to participate in the encounters, veterans quickly returned to well-known dominant narratives. As an example, one of the groups was initially quite satisfied with the encounter they had had, and this satisfaction was shared within the group and yet, a few days later, these feelings seemed to have disappeared. Veteran Martin reflected that: “*The need was for space to express how difficult it is for the soldier. That is almost never possible if you compare [it to] what they have experienced. Fortunately, it turned out well for us, but it did not make much of a difference*.” Old perceptions of Bosnia and Bosnians resurfaced, emphasizing risk and danger—perceptions that were quickly adopted by the group, who stressed that they were “*really lucky that our meeting went well.*” Was the success of the encounters an exception to the rule? A dominant discourse persists—that it is not possible to compare the survivors’ suffering with that of the veterans, Martin exposes his desire to be recognized for his suffering but also realizes this is an improper request in relation to the survivors’ suffering. Still, his remark suggests that he is able to place his own experience within a larger story on Srebrenica, which is a start to thinking relationally.

In the veterans’ return to old discourses, we see just how difficult it is to get rid of dominant narratives of risk and danger. Reverting to familiar images of each other was often easier than creating new narratives. Likewise, Bosnians retreat to discourses about testifying and speaking out. Some reflections do provide an opening, however small this might be, to taking up the slow process of restorying. The focus on peacebuilding and conflict resolution is often about pragmatic solutions created in relation to what is possible within existing dominant narratives. However, “we have rarely engaged ourselves in the deeper search, which requires an imagination that explores narrative as long history, the location of whole peoples’ place in local, national, and global history and as part and parcel of collective healing and the building of justice” (Lederach, 2005, p. 136–7). To truly get somewhere, a much longer investment is needed. Patience with the ‘other’ is necessary.

## Discussion

In this article, we aimed to identify the conditions needed to enable meaningful and even transformative encounters between Dutch veterans and women survivors of the Srebrenica genocide. While dialogue is a dominant frame in peace building endeavours, the encounter is central to this article. This allowed us to explore relations of power and care (Tucker, 2016). Everingham et al. (2021) stated that in post-conflict contexts the success of an encounter is dependent on the willingness of visitors and hosts to think and do differently; that an ‘unlearning’ of earlier experiences acquired perspectives, narratives and worldviews that are needed to establish a more equal relationship which is a precondition for an encounter. Consequently, we explored the spaces those encounters in Bosnia give for storytelling that goes beyond the hegemonic narratives—narratives that do not facilitate understanding but rather reproduce dualistic thinking. We suggest a relational approach in which the experiences of different participants are explored and connected.

Our analysis showed that participants held onto old narrative patterns, often acquired during the war. In the decades that followed the war, these narratives were engrained into collective discourses and memories. Dutch veterans positioned themselves in reaction to societal discourses of political and military failure (e.g., Uittenbogaard, 2024), and highlighted the powerlessness, dangers and traumas they experienced. Bosnian survivors adopted discourses known from other wars and genocides in their search for justice, the recognition of suffering, and fight against genocide denial, in a gendered way (Jacobs, 2017). Both discourses are mirrored in the image participants have created of each other and this impacts on the way conversations unfold.

### Interventions to dominant narratives

Interventions to counter hegemonic narratives were made in multiple forms. Adding the voice of partners to the encounters allowed for different conversations to emerge, and enabled participants see each other in new ways. However, acting in this way as a ‘bridge’ can be a burden to the partner (Driessen et al., 2024). There were moments where survivors and veterans connected through a shared discourse. Recognizing each other in a discourse of shared victimhood permitted individuals to connect across historical, emotional and political divides. Although such a shared discourse might be appealing, allusions to victimhood remain ambiguous (Helms, 2013) and stand in the way of contradictory voices and identities being able to surface, both in the Bosnian and the Dutch case. The institutional aspect of the encounters is a limiting factor: participants become political players, representatives of institutions or organizations. The question is what type of strategies are needed to mitigate for these tensions and to transform the imbalances that emerge in this specific context.

We saw that on an individual level different conversations were possible. Questions addressing situations of humiliation, dehumanization, implication and responsibility become more acceptable when there are just a few people involved. Although these questions might lead to moments of discomfort, we argue that when participants are able to move beyond their comfort zone, they can break down rigid and dominant narratives, and employ the discomfort felt for personal reflection (Soulard et al., 2021). These interventions allow for different stories and contexts to emerge alongside their own more familiar narratives. This is also an opportunity for participants to put their own experiences into perspective. Hence, while the dichotomous memory cultures put Dutch veterans and Bosnian survivors in opposing positions, the encounters we have presented above allow individual participants to recognize that they are both seeking a similar goal and that they have more in common than they thought. Both oppose the institutional neglect of responsibilities that caused so much harm. Witnessing each other’s pain might then help to bring participants closer together (Gobodo-Madikizela, 2016, p. 127).

While many of the encounters are brief and superficial, these small encounters together facilitate a dialogue aimed at mutual understanding. This means it is not about ‘one’ dialogue, but ‘multiple dialogues,’ with multiple participants, and taken together this might ultimately change the way that encounters are talked about and experienced. The realization that the trips are but one step in a much longer process helps to create a better understanding of how this trip fits into the larger trajectory.

To sum up, to make the return trips more beneficial, encounters need to be thought of in a different way; facilitating interventions so that they are not simply ‘incidental’ but that there are careful steps made in how the conversations are structured, and challenging the participants to go beyond their ‘comfort zones.’ Imagining how to go beyond their comfort zones is something that could happen prior to the return trip. It would mean providing a more complex historical narrative about the war, allowing the past to be imagined differently and deliberately creating cracks in a story line that can encourage new ways of thinking about the war before the actual trip, setting a scene for the unexpected and different encounter. This would help participants move more easily away from dominant narratives, prepared and encouraged by institutions to do so. Participants could be encouraged to expand their ‘web of relations’ by including those who are connected to the pain and suffering experienced (Lederach, 2005). Doing so requires more than simply placing two groups together: our research shows that often, a third party is needed to prepare, facilitate and evaluate the encounter. This seems particularly relevant in a memory culture and a military context that is risk averse.

Our research predominantly focused on narrative discourses to demonstrate how premeditated conceptions of ‘the other’ impact on the encounters and how to make sense of these dominant narratives. This approach captures the tensions that surface between past memory and actual experiences of the encounters. It is important to emphasize that even though hegemonic narratives hold a strong power over participants, the move away from them does not necessarily need to be narrative, but can also be a nod, a gift, a hug, or even simply being together. In a context in which participants do not speak each other’s language, such gestures become even more significant. Further research is needed to examine the significance of these gestures in more detail.

This research focused on encounters between two specific groups impacted on by the Bosnian war, the genocide, its legacy, and the longing to ‘unburden us and them.’ Where work on conflict resolution tends to focus on practical solutions and instrumental policies, working with the concepts of the *moral imagination* and *restorying* has allowed us to explore implicit perspectives of ‘othering,’ perspectives that inform us as to how best to prepare for more sustainable connections.

## Recommendations

### Beyond single identities

We formulated three main recommendations that might improve the outcomes of these encounters. First, to encourage discussions with veterans and survivors about their identities that go beyond the stereotypical role of ‘veteran’ or ‘survivor.’ It is important to emphasize the participants on both sides see themselves and the other as a whole person—a parent, a professional, a community member—going beyond the narrower lens that they had in the past. A key for fostering a deeper understanding and a more meaningful connection is the capacity to reach beyond stereotypes. By encouraging individuals to explore and share more nuanced aspects of their identities they are able to see beyond a single and static representation of the conflict they experienced. Veterans may feel limited by the narrow definition of their role during the conflict, while survivors may feel similarly boxed into a victim identity. Encouraging people to talk about the many different aspects of themselves allows for a far more humanizing and relational approach. The onus is also on institutions who need dismantle the one-dimensional narratives, allowing space for complexity, and for developing a critical perspective on their own positionality (e.g., Psaltis et al., 2017).

### Developing theoretical and practical substantiation

Second, the preparation of these encounters requires more theoretical and practical substantiation. Political pressure pushes for a quick fix but for implementing return trips, it is important to engage with experts in peacebuilding and conflict transformation to help achieve set objectives. If institutions were to be more explicit in their communication around the purpose of the encounters, participants might better understand why it is necessary to be open to perspectives that challenge their own. It could be helpful to frame the conversations as opportunities for growth, rather than confrontation.

### Long term engagement and relation building

Third, it is important to develop strategies that maintain engagement and involvement of both survivors and veterans over time. Currently, there is a lack of clarity on how these relationships will be sustained after the initial encounter. One of the challenges with these types of encounters is to ensure that the impact endures beyond the immediate conversations. The recommendation is to establish strategies for follow-up and continued interaction, whether through regular meetings, support groups, or continued collaboration on shared projects (e.g., memorial events or community initiatives). Without these, the connections made during the encounters are likely to fade over time. Creating a structure for sustained engagement will help ensure that the relationships and new understandings that are being built through the dialogues, continue to evolve.

## Data Availability

The datasets presented in this article are not readily available because this study focuses on a very specific group of people, that might be identifiable even in an anonymized dataset because of the context that such a dataset provides. Requests to access the datasets should be directed to SD s.driessen@uvh.nl.

## Footnotes

^1^Although individual veterans have been returning to Srebrenica for a longer period (see Driessen, 2021), organized encounters between survivors and veterans have been rare. Since the institutionalization of the return trip, the encounters have become an obligatory part of the program – they are both a precondition set out by the Potočari memorial for veterans who visit Srebrenica and a conviction of the Dutch and Bosnian organizers that contact with survivors will benefit veterans and vice versa (see Vermetten et al., 2024). Nevertheless, among participants the purpose and rationale of the meetings is often quite unclear.

^2^It is important to recognize that genocide does not start with mass killings – which are evidence of the final phases of a much longer process that starts with for instance (ethnic) classification and dehumanization (see for instance Stanton, 2023) and the 10 stages of genocide, https://www.genocidewatch.com/tenstages; Karčić (2022) *Torture, Humiliate, Kill* p. 30.

^3^The focus on the consequences of the deployment to Srebrenica for veterans implies that in the Netherlands, the attention to the experiences of genocide survivors and relatives of victims remains scarce (Mustafić and Wentholt, 2025).

^4^Some examples: Tea Tupajić’s plays *Dark numbers* and *Darkness there and nothing more*, Alma’s Mustafić’s and Boy Jonkergouw’s play *Dangerous names*; Bosnian girl’s *Temporary Monument: Srebrenica is Dutch history too;* Nick Teunissen’s play *One way ticket*; the exhibition at the Potočari memorial, co-created by Bosnia and the Netherlands; the ‘Facing Srebrenica’ project in which veterans return pictures they took in Srebrenica to the Potočari Memorial Centre.

^5^The main part of fieldwork took place January–September 2023. After this period, we attended evaluation meetings more sporadically. The authors’ collaboration on researching return trips started in 2020.

^6^From the Dutch side, the trips (and meetings) are supervised by military chaplains and social workers. Traumatized veterans are usually already involved in treatment in the Netherlands – their therapists will likely assess their participation in the return trip. Snaga Žene provides psychological care to its members and supervisors are present during the meetings with veterans. Nevertheless, despite supervision, it is important to note that there is the possibility that participants might experience harm.

^7^For a more extensive discussion of gender dynamics in studying the military, see Driessen et al., 2024.

^8^All names are pseudonyms.

^9^For a more elaborate discussion of partners’ role during the return trip, see Driessen et al., 2024.
